# Prevalent vertebral fractures among urban-dwelling Chinese postmenopausal women: a population-based, randomized-sampling, cross-sectional study

**DOI:** 10.1007/s11657-022-01158-x

**Published:** 2022-09-07

**Authors:** Weibo Xia, Qiang Liu, Jinhan Lv, Zhenlin Zhang, Wen Wu, Zhongjian Xie, Jianting Chen, Liang He, Jian Dong, Zhenming Hu, Qiang Lin, Wei Yu, Fang Wei, Jue Wang

**Affiliations:** 1 Department of Endocrinology, Key Laboratory of Endocrinology, National Commission of Health, State Key Laboratory of Complex Severe and Rare Diseases, Peking Union Medical College Hospital, Chinese Academy of Medical Sciences, Beijing, China; 2 Shanxi Dayi Hospital, Taiyuan, Shanxi China; 3 The People’s Hospital of Ningxia Hui Autonomous Region, Yinchuan, Ningxia China; 4 Department of Osteoporosis and Bone Disease, The Sixth People’s Hospital, Shanghai Jiaotong University, Shanghai, China; 5 Guangdong Provincial People’s Hospital, Guangzhou, Guangdong China; 6 National Clinical Research Center for Metabolic Diseases, Hunan Provincial Key Laboratory of Metabolic Bone Diseases, and Department of Metabolism and Endocrinology, The Second Xiangya Hospital, Central South University, 139 Middle Renmin Road, Changsha, Hunan China; 7 Division of Spine Surgery, Department of Orthopaedics, Nanfang Hospital, Southern Medical University, Guangzhou, China; 8 Beijing Jishuitan Hospital, Beijing, China; 9 Fudan University Zhongshan Hospital, Shanghai, China; 10 The First Affiliated Hospital of Chongqing Medical University, Chongqing, China; 11 Department of Radiology, Peking Union Medical College Hospital, Chinese Academy of Medical Science, Beijing, China; 12 Medical Affairs & Outcomes Research, Organon China, Shanghai, China; 13 Global Medical and Scientific Affairs, Merck Research Laboratories, MSD China, Shanghai, China

**Keywords:** Prevalent vertebral fractures, Osteoporosis, Randomized sampling, Postmenopausal women, Community-dwelling

## Abstract

**Summary:**

In this population-based, cross-sectional study, we investigated vertebral fracture (VF) prevalence among Chinese postmenopausal women. We found 14.7% of population had VFs, which increased with age. Age ≥ 65 years, hip fracture, and densitometric osteoporosis were significantly associated with VFs. The prevalence of osteoporosis was remarkably high.

**Purpose:**

To investigate VF prevalence among Chinese postmenopausal women in this population-based, randomized-sampling, cross-sectional study.

**Methods:**

The investigator obtained lists of women from communities. Randomization was performed using SAS programming based on age group in each region. Postmenopausal women aged ≥ 50 years in the urban community were included. The investigator interviewed subjects to collect self-reported data and measured BMD. Spine radiographs were adjudicated by Genant’s semi-quantitative method. VFs were defined as fractures of at least one vertebra classified by Genant’s score 1–3 and were analyzed using descriptive statistics.

**Results:**

A total of 31,205 women listed for randomized sampling from 10 Tier-3 hospitals at 5 regions. Of 2634 women in the full analysis set, 14.7% (388/2634, 95% CI: 13.4, 17.1) had prevalent VFs. VF prevalence increased with age (Cochran–Armitage test *p* < 0.0001) and was significantly higher in women aged ≥ 65. VF prevalence did not differ between North (14.4%, 95% CI: 12.5, 16.4) and South China (15.1%, 95% CI: 13.3, 17.1). In women with no prior VFs, prevalent VFs were 12.4% (95% CI: 11.2, 13.7). Age ≥ 65 years (OR: 2.57, 95% CI: 1.91, 3.48), hip fracture (OR: 2.28, 95% CI: 1.09, 4.76), and densitometric osteoporosis (OR: 2.52, 95% CI: 1.96, 3.22) were significantly associated with prevalent VFs. Prevalence of osteoporosis was 32.9% measured by BMD and 40.8% using NOF/IOF clinical diagnosis criteria.

**Conclusion:**

VFs are prevalent among Chinese postmenopausal women who were ≥ 50 years and community-dwelled. Osteoporosis prevalence is remarkable when fragile fractures were part of clinical diagnosis.

**Supplementary Information:**

The online version contains supplementary material available at 10.1007/s11657-022-01158-x.

## Introduction

Osteoporosis is prevalent worldwide [1] and causes fractures in both men [2] and women [3]. Osteoporotic fractures lead to subsequent fractures [4], loss of quality of life [5, 6], and excess risks of hospitalization and mortality [7]. Vertebral fractures (VFs) are the most common manifestation of osteoporosis. VFs predict a future fracture at any skeletal site independent of BMD [4, 8] and are associated with poor quality of life [9] and mortality [10]. The risks of VFs are population-specific on different lifestyles, environments, medical care, and ethnicities [11]. Treatments to risk factors prevent future fractures [8, 12]; however, most VFs are asymptomatic and receive little clinical attention. A qualitative or quantitative radiologic approach is recommended to assess VFs. However, a substantial proportion of VFs may remain undiagnosed at the time of occurrence or even during the lifetime [13, 14].

Vertebral fractures increase with age among postmenopausal women. Published literature has suggested an overall prevalence between 10–20% [15–20] and 35–40% in women aged 80 years or older [20–22]. Unlike hip fractures, prevalent VFs appeared similar between Caucasian and Asian women [21–23]. Population-based studies investigating prevalent VFs and risk factors in China began in the early 1990s but were mainly conducted in a single city. Observational studies in Beijing [20, 24], Hong Kong [22, 25], and Shanghai [26] showed rates of prevalent VFs ranging between 15 and 23% among Chinese postmenopausal women. These studies, however, did not show an increasing trend in prevalent VFs among postmenopausal women over time, in contrast to a 3–fourfold increase in prevalent hip fracture in the same population [27]. Therefore, we aimed at investigating the age-specific prevalence of radiographic VFs among postmenopausal women by a population-based study in China. Second, we observed prevalent osteoporosis and determined the risk factors for prevalent VFs.

## Materials and methods

### Study design and setting

This was a population-based, randomized sampling, cross-sectional study investigating the prevalence of VFs among urban-dwelling postmenopausal women in China (China Vertebral and Osteoporosis Study, ChiVOS). The study selected 5 geographic regions (East, West, South, North, and Central) in China to recruit postmenopausal women. Subject enrollment commenced in January 2017 and ended in July 2018 at outpatient endocrinology or orthopedics clinics from 10 Tier-3 hospitals in 5 geographic regions (Supplementary Fig. 1). Merck Sharp & Dohme (MSD) China and leading principal investigators designed the study and analyzed the data. The study followed the International Conference on Harmonization and Good Pharmacoepidemiology Practice (GPP), the Declaration of Helsinki, and applicable local regulatory guidance. Independent ethics committees of all sites approved the study before any study-related procedure.

### Randomized sampling and participants

The study used a two-stage cluster sampling approach to enroll subjects. Firstly, 5 geographic regions were identified as the cluster. The study team determined 2 participating sites out of 8–10 Tier-3 hospitals in each cluster based on expertise and study resource. Randomization was not performed for site selection due to operational difficulties. At the second stage, the investigator at each site obtained lists of community-dwelling women from community-dwelling and primary care health records. The lists only extracted demographic information. The investigator selected female residents aged ≥ 50 years, numbered them by age group (stratum), masked all identifiable bio-information, and presented them to the study statistician who generated a new list to resequence residents by stratum using SAS randomization programming. The investigator used the original resident number retained in the randomization list to find the residents’ contact details to invite them by post, phone calls, or other electronic methods. The resident who accepted the invitation was asked to undergo on-site formal study screening procedures.

The investigator assessed the subject eligibility at the site. Women were eligible if they were Chinese aged 50 years or above, postmenopausal, willing to consent, and lived in an urban community > 6 months. Menopause was defined as no menses naturally for at least a year by self-reporting, or 6 months of spontaneous amenorrhea with serum FSH levels > 40 mIU/mL, or at least 6 months after a surgical bilateral oophorectomy with or without hysterectomy. Women were excluded if they were not Asian and had cognitive impairment (if no legal representative), physical impediment, or other potential reasons (i.e., non-compliance) affecting the completion of the study procedure. The recruitment was independent of a seasonal or social impact and planned to occur at any time point. All subjects or their legal representatives provided written informed consent before any study screening procedure.

### Study procedure and clinical assessment

The subject paid a single visit to the site for all study-related procedures. Upon inclusion, the investigator performed an interviewer-administered questionnaire. The subject was required to have BMD measurement and vertebral radiologic assessment after the completion of the interview. The investigator may perform a physical examination for fracture-related medical history whenever necessary.

#### Questionnaire

The questionnaire was interviewer-administered and designed based on previous studies [28–33] with the local language and cultural and clinical adaptions (Supplemental sample questionnaire). One (1) designated investigator performed questionnaire interviews for all subjects at each site to minimize variability in data collection. The questionnaire collected the subject’s self-reported demographics, socioeconomic status, lifestyle, and clinical characteristics for osteoporosis and fragile fractures.

#### Bone mineral density

A dual-energy x-ray absorptiometry (DXA) device (Hologic, Lunar or Norland) measured BMD at each subject’s lumbar spine, the femoral neck, and the total hip. The study did not allow a DXA measurement on a mobile health van. Quality control and calibration of DXA machines (i.e., coefficient of variation < 3%) were based on the site routine practice as recommended by the manufacturer (GE Lunar and Hologic) at each site. BMD measurement was done by the same machine or machine type from the manufacturer for all subjects at each site.

All sites used the same scanning protocol on patient positioning. Patient was positioned straight on the DXA table and centered in the field with no rotation. The spine can be shown straight (spinous processes are centered with soft tissue equal on either side) on the image from the testing report (spine is straight on the image). The scan included at least the lowest vertebra with ribs (T12) and L4 and L5. The investigators reviewed the DXA BMD report to check the patient positioning and scanning area.

#### Vertebral radiologic imaging and VF adjudication

The site’s radiologists followed the study protocol to perform lateral radiographs centered on the thoracic (T)7 and the lumbar (L)2 vertebrae. Radiographs were taken with the subject in the left lateral position by breathing to blur the overlying ribs and lung by motion. The investigator sent radiographs to the central adjudication team (Peking Union Medical College Hospital), who standardized fracture assessment using Genant’s semi-quantitative method with excellent inter- and intra-observer agreements [34]. Two (2) professorial radiologists independently inspected qualitative vertebral shape and degree of reduction in vertebral height in the anterior, middle, or posterior vertical dimension. A vertebral body was graded as normal or characterized by a mild, moderate, or severe fracture. The adjudication and the study teams reviewed the subject’s medical history for causes of VFs other than osteoporosis to exclude subjects who had non-osteoporotic VFs (i.e., trauma or cancer bone metastasis).

### Outcome measures

#### Vertebral fracture

A vertebral fracture is defined as the presence of at least one vertebra morphometrically classified as grade 1 (mild), 2 (moderate), or 3 (severe) on Genant’s score [34] for vertebral fracture as agreed by both radiologists from the central adjudication team. The Genant’s definitions are 20–25% reduction in anterior, middle, and/or posterior vertebral height and 10–20% in area was defined as grade 1 deformity; a 25–40% reduction in any height and area was defined as grade 2 deformity, and a 40% reduction in any height and area was regarded as grade 3 deformity.

#### Osteoporosis

Densitometric osteoporosis is defined as a BMD T-score ≤  − 2.5 in at least one of the anatomic sites, including the lumbar spine, the femoral neck, and the total hip [28] (WHO). The study also defined clinical diagnosis of osteoporosis using recommendations from the International Osteoporosis Foundation/National Osteoporosis Foundation (IOF/NOF) (2014) [29], the Chinese Society of Bone and Mineral Research (CSOBMR) [30], and the American Association of Clinical Endocrinology (AACE) (2020) [31].

#### Fragile fracture

A fragile fracture is defined as a fracture that occurred with minimal trauma at a typical site of osteoporotic fracture that would be unlikely to cause a fracture in a non-osteoporotic adult [32, 33].

### Statistical analysis

#### Sample size consideration

The sample size was calculated based on previous prevalence data per age group [15–17, 20]. The sample size was determined by age group setting a lower limit for each assumed age-based prevalence (Supplemental Table 1). Clopper-Pearson exact method [35] was used to compute the lower bound of 95% confidence interval (CI) at a 5% precision for the point estimate. A target sample size of 545 was statistically sufficient to estimate the age-specific VF prevalence at each region, assuming 5% of withdrawals from the on-site radiologic procedures. A total sample size of approximately 2700 postmenopausal women was planned. This sample size also yielded a 95% power at a 2-sided significance level of 0.05 to detect a geographic impact for VF prevalence by an odd ratio at 1.40 or 1.50 in a univariate logistic model.

#### Study objectives and analysis population

Descriptive statistics addressed the analysis of prevalence in VFs as the primary objective. Percentages of prevalence with a two-sided 95% CI were calculated in overall study population, age groups, and geographic regions. The 95% CI calculation used a more conservative Wilson Score method [35, 36]. The analysis stratified geographic region by a general division (East, West, South, North, and Central) and a specific definition using Qinling Mountains-Huaihe River Line (North versus South China).

The statistical analysis plan (SAP) specified all variables (risk factors) associated with prevalent VFs among postmenopausal women. A risk factor with a p-value ≤ 0.1 in univariate logistic regression entered the multivariate logistic regression model (two-step statistical modeling). The multivariate analysis gave an adjusted OR and corresponding 95% CI for each risk factor in the model. A ROC (Receiver Operating Characteristics) AUC (Area Under the Curve) showed the model prediction strength for prevalent VFs.

The full analysis set (FAS) included all subjects who had vertebral radiologic procedures in the study. The per protocol set (PP) included all subjects who had per-protocol vertebral radiologic procedures in the study. Subjects who did not complete both thoracic and lumbar radiologic assessments were excluded from the PP. The FAS serves the analysis population for the primary objective.

The statistical analysis did not impute missing data. All analyses were performed by using SAS 9.4, SAS JMP, and 15.2 (SAS Institute, Cary, NC, USA). A p-value of 0.05 or less at 2-sided significance was considered statistically significant whenever applicable. Statistical comparisons on prevalence were made using data visualization. No overlap between the two 95% CIs for prevalence concluded a significant difference.

## Results

### Subject disposition and characteristics

**Fig. 1:**
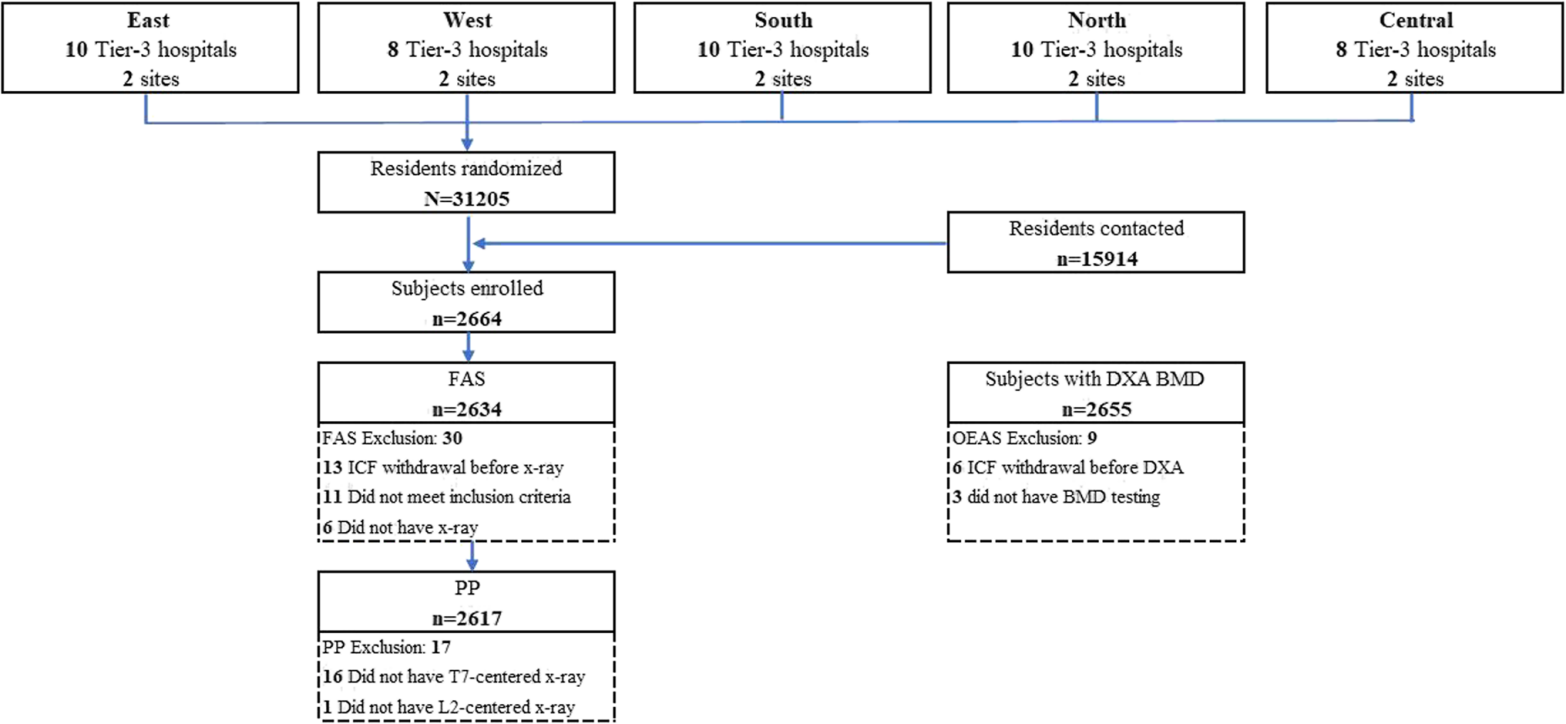
Study flow for subject disposition and analysis population. Five (5) geographic regions were identified as the cluster; 2 sites out of 8–10 Tier-3 hospitals in each cluster were selected. The investigator at each site obtained lists of community-dwelling women as residents with extracted demographic information for randomized sampling procedure done by a study statistician using SAS programming. The investigator contacted residents. Women may have been excluded from one analysis population for more than one reason, but were counted in one outstanding reason leading to exclusion. FAS: full analysis set; PP: per protocol

Figure 1 shows the randomization, subject enrollment, and analysis populations. The investigators obtained resident lists containing 31,205 community-dwelling women from 5 regions across China. Of 2664 women enrolled, 2634 (98.9%) and 2617 (98.2%) were in the FAS and PP, and 2655 had BMD measurements on-site (99.7%). The most frequent reason leading to the exclusion from the FAS was ICF withdrawal (the subject withdrew the consent to the study) before radiologic imaging (13/30, 36.7%).

Table 1 displays the subject’s demographic and clinical characteristics in the FAS. The mean age (SD) of all women was 66.9 (8.8) years. The mean age of women who had prevalent VFs was numerically higher than that of those who did not. Women at high risk of osteoporosis were 17.7% (465/2634) assessed by Osteoporosis Self-Assessment Tool for Asians (OSTA). Women who were smoking were 2.2% (58/2634). Women who had taken daily calcium (23.3%) and daily vitamin D (12.8%) were 23.3% and 12.8%, respectively. In the FAS, 19.7% (520/2634) of women experienced at least one fracture after 50 years of age; 20.0% (527/2634) of women reported osteoporosis diagnosis. The use of antiresorptive agents was relatively low (134/2634, 5.1%).

**Table 1:** Demographic and baseline characteristics (FAS)

	Genant’s radiographic assessment				All	
	Normal		Vertebral fracture			
	*N* = 2246		*N* = 388		*N* = 2634	
Age	65.9	8.6	72.7	7.8	66.9	8.8
≥ 65 years	1156	51.5	319	82.8	1475	56.0
BMI	24.6	3.6	24.9	3.5	24.6	3.6
≥ 30 kg/m^2^	162	7.2	40	10.4	202	7.7
< 20 kg/m^2^	192	8.7	29	7.7	8.5	221.0
OSTA risk category						
Low (> − 1)	1099	48.9	103	26.5	1202	45.6
Medium (− 1 to − 4)	816	36.3	151	38.9	967	36.7
High (< − 4)	331	14.7	134	34.5	465	17.7
Social status						
Higher education ^a^	354	15.8	55	14.2	409	15.5
Dominantly labor work	1092	48.6	213	54.9	1305	49.5
Family low income ^b^	114	5.1	29	7.5	143	5.4
Life style						
Current smoker ^c^	48	2.1	10	2.6	58	2.2
Calcium daily	512	22.8	102	26.3	614	23.3
Vitamin D daily	273	12.2	63	16.2	336	12.8
Exposure to sunshine	349	15.5	83	21.4	432	16.4
Outdoor exercise (> 1 h)	734	32.9	123	31.9	857	32.7
Self-reported medical history						
Hypertension	771	34.6	181	46.9	952	36.4
Diabetes mellitus	372	16.6	66	17.0	438	16.6
Cancer history	77	3.4	18	4.6	95	3.6
Osteoporosis	383	17.1	144	37.1	527	20.0
Use of antiresorptive drugs	89	4.0	45	11.6	134	5.1
Fall on the flat ground ^d^	588	26.2	186	47.9	774	29.4
Fracture after 50 years	355	15.9	164	42.5	519	19.8

Data are presented by mean + / − SD or N and %; ^a^college or above education received; ^b^income less than CNY 1000 per month; ^c^at least one cigarette everyday for 6 months or above; ^d^fall on the flat ground after 50 years old; *FAS*, full analysis set; *BMI*, body mass index; *OSTA*, Osteoporosis Self-Assessment Tool for Asians

### Prevalence of vertebral fractures

Table 2 exhibits age-specific prevalent VFs. The prevalence of VFs standardized by Genant’s semi-quantitative method was 14.7% (388/2634, 95% CI: 13.4, 16.1) among Chinese postmenopausal women. The prevalence of VFs was significantly higher among women who were ≥ 65 (21.6%, 319/1475, 95% CI: 19.5, 23.7) compared with those who were < 65 (6.0%, 69/1159, 95% CI: 4.6, 7.3). Prevalent VFs increased with age (Cochran Armitage trend test *p* < 0.0001). In women who were ≥ 80 years of age, VF prevalence was 35.7% (95% CI: 29.6, 41.7).

**Table 2:** Age-specific prevalent vertebral fractures assessed by Genant’s semi-quantitative method (FAS)

Crude prevalence ^a^	Vertebral fracture			
		Genant’s semi-quantitative method		
	n	N	%	95% CI ^b^
Overall	388	2634	14.7	13.4, 16.1
Age group				
** < **65 years	69	1159	6.0	4.6, 7.3
50–54	2	220	0.9	0.0, 2.2
55–59	17	408	4.2	2.2, 6.1
60–64	50	531	9.4	6.9, 11.9
** > = **65 years	319	1475	21.6	19.5, 23.7
65–69	61	460	13.3	10.2, 16.4
70–74	87	417	20.9	17.0, 24.8
75–79	84	354	23.7	19.3, 28.2
80 +	87	244	35.7	29.6, 41.7

^a^Categorical data are expressed as n and %; ^b^95% CI is calculated using Wilson score method. Vertebral fracture is defined as the presence of fracture in at least one vertebra classified by Genant’s score 1, 2, or 3 as adjudicated by two radiologists. *FAS*, full analysis set; *CI*, confidence interval

**Fig. 2:**
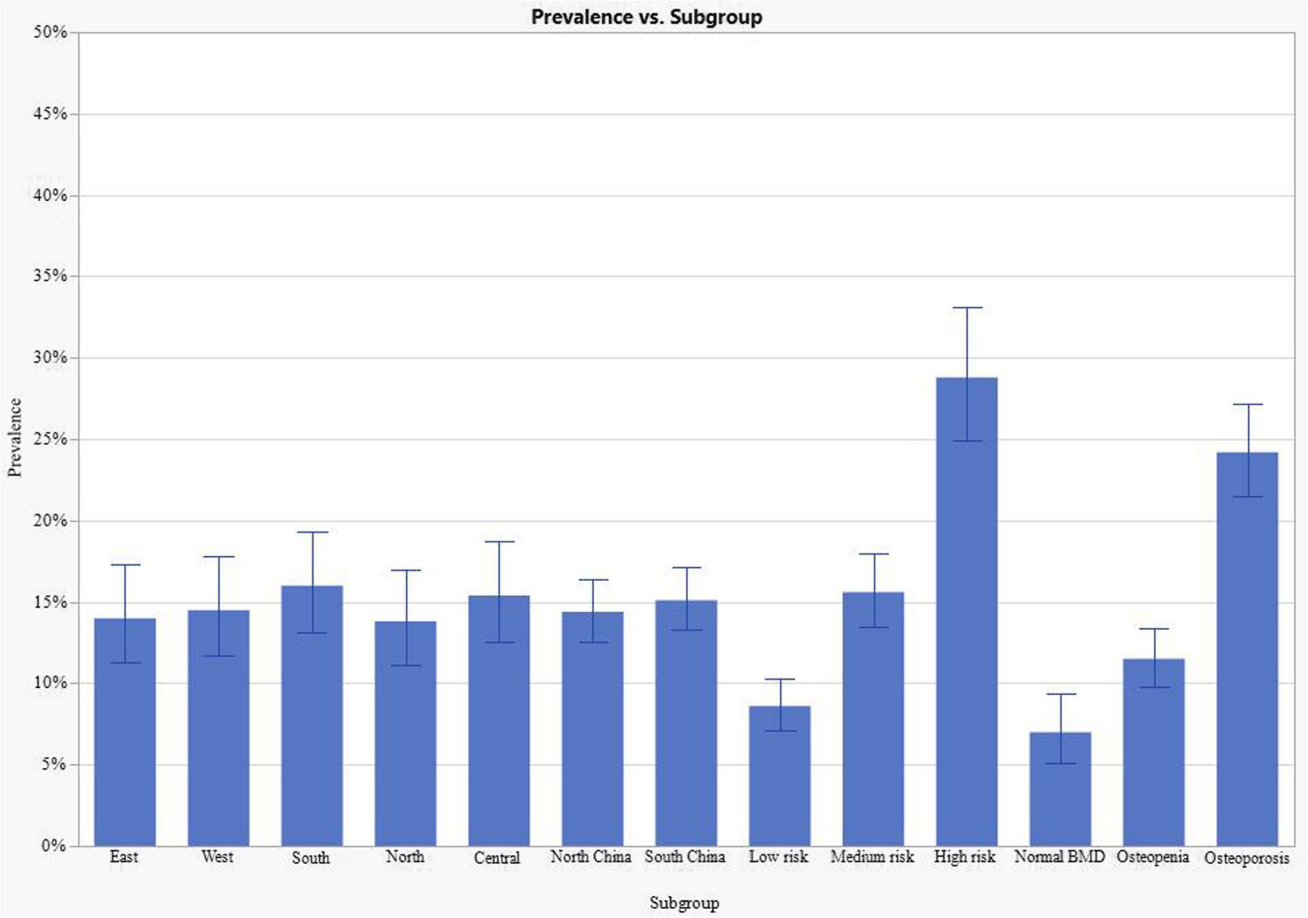
Prevalence of vertebral fractures by subgroup. Data are shown as point estimate (prevalence) and 95% CI. Prevalence of vertebral fractures was analyzed in the subgroups of geographic regions, risk of osteoporosis by OSTA, and BMD measured by DXA

Figure 2 shows the prevalence of VFs by subgroups. There was no significant difference in the prevalence of VFs among 5 prespecified geographic regions. Prevalence did not differ significantly between women who lived in North (182/1268, 14.4%, 95% CI: 12.5, 16.4) and South China (206/1366, 15.1%, 95% CI: 13.3, 17.1). The prevalence of VFs increased significantly with decreased BMD among postmenopausal women (Cochrane Armitage trend test *p* < 0.01). In women who had densitometric osteoporosis, prevalent VFs accounted for 24.2% (210/868, 95% CI: 21.5, 27.2); in women who had normal BMD, prevalent VFs accounted for 7.0% (38/545, 95% CI: 5.1%, 9.4).

### Clinical features of prevalent vertebral fractures

Table 3 presents clinical features of prevalent VFs. The proportion of severe (90/388, 23.2%) or multiple fractures (62/388, 16.0%) was relatively lower among all women who had VFs. Thoracic VFs presented in 62.4% (242/388) of women. Among women who had a history of any fragile fracture, prevalent VFs were 31.5% (164/520, 95% CI: 27.7, 35.7). In women who did not report prior VFs, prevalent VFs were 12.4% (314/2533, 95% CI: 11.2, 13.7). Of note, prevalent VFs accounted for 44.1% (25/34, 95% CI: 28.9, 60.6) of women who reported prior hip fractures. In women with no NVNH (non-vertebral non-hip) fractures, prevalent VFs were 14.0% (314/2246. 95% CI: 12.6, 15.5).

**Table 3:** Clinical features of prevalent vertebral fractures (FAS)

	Vertebral fracture ^a^			
		Genant’s semi-quantitative		
	n	N	%	95% CI
Severity ^c^				
*Mild*	193	388	49.7	44.8, 54.7
*Moderate*	105	388	27.1	22.6, 31.5
*Severe*	90	388	23.2	19.0, 27.4
Fracture type ^d^				
*Mono fracture*	326	388	84.0	80.4, 87.7
*Multiple fractures*	62	388	16.0	12.3, 19.6
Fracture location				
*Thoracic*	242	388	62.4	57.6, 67.2
*Lumbar*	208	388	53.6	48.7, 58.6
Fall after 50 years old				
*Yes*	186	774	24.0	21.2, 27.2
*No*	202	1860	10.9	9.5, 12.4
Self-reported osteoporosis				
*Yes*	144	527	27.3	23.7, 31.3
*No*	244	2107	11.6	10.3, 13.0
Any prior fragile fractures ^e^				
*Yes*	164	520	31.5	27.7, 35.7
*No*	224	2114	10.6	9.4, 12.0
Prior vertebral fractures				
*Yes*	74	101	73.3	63.9, 80.9
*No*	314	2533	12.4	11.2, 13.7
Prior hip fractures				
*Yes*	15	34	44.1	28.9, 60.6
*No*	373	2600	14.4	13.1, 15.8
Prior NVNHFX				
*Yes*	74	388	19.1	15.5, 23.3
*No*	314	2246	14.0	12.6, 15.5

^a^Categorical data are expressed as n, N, and %; the subject with vertebral fractures is defined as the subject presented by at least one vertebra classified by Genant’s grade 1–3 as suggested by both radiologists; ^b^95% CI is calculated using Wilson score method; ^c^for subjects who had multiple vertebral fractures, the highest Genant’s grade is used to count the severity; ^d^mutliple fractures refer to fractures in two or more vertebral locations; ^e^all prior fractures were based on the subject’s self-reporting during the investigator-administered questionnaire interview; *FAS*, full analysis set; *NVNHFX*, non-vertebral non-hip fragile fractures; *CI*, confidence interval

### Prevalence of osteoporosis

The prevalence of densitometric osteoporosis was 32.9% (874/2655, 95% CI: 31.1, 34.7) among Chinese postmenopausal women. Women who were ≥ 65 years (42.5%), who lived in South China (38.9%), and who had medium (39.1%) and high (66.0%) risks of osteoporosis defined by OSTA had significantly higher prevalence of densitometric osteoporosis (no overlaps between 95% CIs to compare references). Prevalent osteoporosis was high using NOF/IOF (46.8%, 1243/2665, 95% CI: 44.9, 48.7), CSOBMR (40.8%, 1084/2655, 95% CI: 39.0, 42.7), or AACE (48.2%, 1279/2655, 95% CI: 46.3, 50.1) clinical diagnosis criteria. This trend in prevalent osteoporosis was similar in the subgroups. Supplementary Table 2 displays all results for prevalent osteoporosis.

### Risk factors for prevalent vertebral fractures

**Fig. 3:**
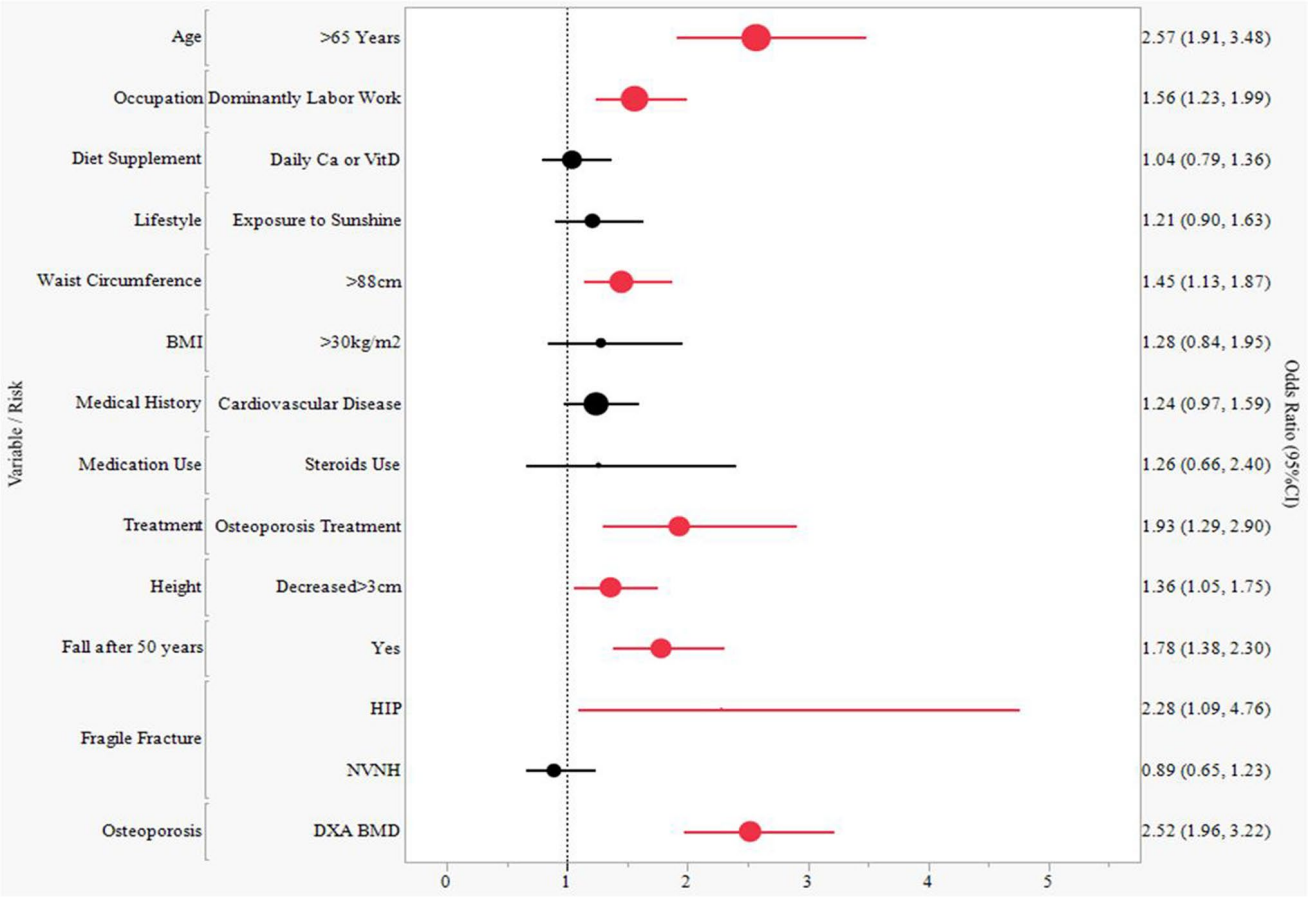
Risk factors associated with prevalent vertebral fractures. All risk factors (independent variable) are binary for vertebral fractures odds of yes versus no; 95% CI is based on normal approximations; age as continuous variable and age groups as ordinal variable are not included in multivariate analysis; infomration on osteoporosis treatment is given by subjects who interviewed the investigators; NVNH: non-vertebral non-hip; CI: confidence interval

In the multivariate logistic model (Fig. 3), adjusted for other variables, age ≥ 65 years (OR: 2.57, 95% CI: 1.91, 3.48 *p* < 0.0001), dominantly labor work (OR: 1.56, 95% CI: 1.23, 1.99, *p* < 0.001), height decreased (OR: 1.36, 95% CI: 1.05, 1.75, *p* = 0.02), fall on the flat ground after 50 years old (OR: 1.78, 95% CI: 1.38, 2.30, *p* < 0.0001), hip fracture (OR: 2.28, 95% CI: 1.09, 4.76, *p* = 0.03), and densitometric osteoporosis (OR: 2.52, 95% CI: 1.96, 3.22, *p* < 0.0001) were associated with an increased risk of prevalent VFs. Women who received osteoporosis treatment had a 1.93-fold likelihood of having a prevalent VF (OR: 1.93, 95% CI: 1.29, 2.90, *p* = 0.002). The multivariate logistic regression exihibited a good model fit of all independent variables (*p* = 0.92). A complete analysis of risk factors associated with prevalent VFs using a two-step logistic regression model is displayed in Supplementary Table 3 with model discriminative capability ROC AUC (0.7496, Supplementary Fig. 2).

## Discussion

Findings from this population-based, randomized-sampling, cross-sectional study suggested VFs were prevalent and increased with age among Chinese postmenopausal women who were 50 years or above and community-dwelling. The study did not suggest a geographic difference in prevalent VFs. The prevalence of osteoporosis was remarkably high among Chinese postmenopausal women.

The study obtained lists of 31,205 residents from local communities to randomize and sample by geographic region and age group. The study adopted an interviewer-administered questionnaire, had BMD measurements at three anatomical sites, used a standard X-ray protocol, and adjudicated all vertebral fractures. This study is among the few Chinese population-based studies on osteoporotic VFs and has been the only nationwide study. The study has limitations. Incident vertebral fractures were not studied because of a cross-sectional design. The analysis of the temporal relationship between osteoporosis and VFs was not feasible. Randomized sampling was performed to recruit women free of volunteer effect. Nevertheless, this procedure inherently brought non-response to study invitation, which was higher than in a previous study in Beijing [20]. The higher non-response rate may be caused by the difference in resource allocations among sites across China. For instance, study information distribution in the community and the follow-up of initial non-responders. The investigator encouraged women to give study-related information during the interview. However, recall bias existed because the interview collected comprehensive social, lifestyle, and clinical information in a relatively short duration. Last, study results are not generalizable to specific patient groups in the community.

The study investigated VF prevalence as the primary objective. Most women (98.9%) followed protocol-specified procedures for radiologic imaging. Study analysis indicated that 14.7% of postmenopausal women had prevalent vertebral fractures. In women with no history of fragile or vertebral fractures, prevalent VFs did not differ significantly compared to the overall population. The prevalence increased with age, in which the highest (35.7%) was observed in women who were 80 years or above. The overall and age-specific VF prevalence was generally comparable to results from studies in the USA [37] and different Asian cities, including Beijing [20], Hong Kong [22, 25], and Shanghai [26]. However, the study design differed between our study and these published studies. US study used DXA-based VF assessment, and studies in Hong Kong and Shanghai did not perform randomized sampling. A recent epidemiological study conducted in Beijing had a similar study design. The overall (23.9%) and age-specific prevalence was higher [24]. One Spanish study also observed an increased prevalence of VFs among postmenopausal women, and the authors believed that women who had malnutrition in hard times between the 1930s and 1950s were recruited [38]. Our study had the same fracture assessment method as the recent Beijing study. Women born in the 1930s and the 1950s were recruited to the age groups between 65 and 80 + . Prevalence from these age groups did not differ significantly from other studies [22, 25, 26]. The observed difference in prevalent VFs between our study and other recent studies is hard to explain and may need further studies to support. Despite this, our study findings on age-specific prevalence (95% CI upper bound 11.3% for age subgroup 60–64) supported the VF screening among postmenopausal women older than 60 in China.

Published studies supported the difference in prevalent VFs among countries [15–20]. One study compared the prevalence in four Asian countries using a standardized fracture assessment method [39]. The prevalence rate in Japan was the highest but did not differ significantly from the current study. Because of China’s geographic size, no study compared prevalent VFs among China’s geographic regions. Our findings suggested no geographic difference in prevalent VFs across China, addressing the uncertainty in geographic prevalence in this big country.

Vertebral fractures are clinically asymptomatic and usually require a radiologic diagnosis. Specific radiologic assessments, including quantitative or semi-quantitative methods, are time-consuming, and trained radiologists are needed. In routine practice, clinical diagnosis missed approximately three-fourths of vertebral fractures in East Asia [40, 41]. This study found a similar situation. The site’s radiologic assessment identified 46.1% of all prevalent vertebral fractures. In light of these results, there is still a possibility that > 50% of vertebral fractures would be missed clinically among postmenopausal women in the real-world clinical setting.

Many studies have demonstrated the high prevalence of osteoporosis in the USA and Europe, and Asian countries, including China [1]. Treatment guidelines from NOF/IOF [29]^(30)^ and AACE [30] incorporated clinical components predicting fracture risks to diagnose osteoporosis. The Chinese Medical Association and CSOBMR applied similar criteria in 2017 [30]. In our study, osteoporosis prevalence increased dramatically among postmenopausal women when NOF/IOF or AACE criteria were applied in the analysis. The results indicated that the osteoporosis prevalence in the real-world setting significantly increased using these recent diagnosis criteria compared to BMD alone. Antiosteoporosis therapy aims at preventing fragile fractures as the primary outcome. Including patients with existing fragile fractures or fracture risks as osteoporotic patients will bring remarkable treatment effects beyond targeting BMD values.

Women enrolled in the study have medical history representing the general Chinese women population. They were generally healthy, had a low proportion of smoking or drinking, had outdoor exercise, and had low calcium and vitamin D intake [24, 26, 42]. Except for the nature of the occupation, lifestyle characteristics were not statistically significant predictors for prevalent VFs. These results were consistent with previous Chinese studies [20, 22, 24–26]. Risk factors were mainly bone health–related, including a prior hip fracture, height decrease, osteoporosis treatment, densitometric osteoporosis, and fall on the flat ground. An interesting finding was the osteoporosis treatment as a risk factor for prevalent VF. This cross-sectional study did not collect data on the temporal relationship between osteoporosis treatment and VFs. Women with prevalent VFs had more osteoporosis treatments than those without prevalent VFs. Therefore, as analyzed, an adverse association between the treatment and prevalent VFs occurred. Also, type 2 diabetes was not significantly associated with prevalent VFs. Other studies reported conflict results—an increased [43] or reduced [44] risk of prevalent vertebral fractures. BMI > 30 kg/m^2^ showed a moderate association with the increased risk of prevalent VFs, which differed from previous findings [45]. Last, we were unable to show daily intake of calcium or vitamin D or exposure to sunshine was associated with reduced VF risks in the multivariate logistic regression. The study did not collect details of calcium or vitamin D intake and cannot verify if the subjects gave the correct estimate of sunshine exposure. Therefore, temporal relationship between VF and appropriate doses of diet supplements or correct life style was not assessed. Moreover, the subject’s recall bias existed in questionnaire-based data collection. Weak beneficial effects associated with VF prevalence from calcium or vitamin D intake or exposure to sunshine can be seen in multivariate analysis when other stronger factors (i.e., age or BMD) are adjusted.

In conclusion, vertebral fractures are prevalent among postmenopausal women who were 50 years or above and community-dwelled in China. The prevalence of osteoporosis is remarkably high among postmenopausal women when major fragile fractures were part of the clinical diagnosis as recommended by NOF/IOF, CMA/CSOBMR, and AACE.

## Supplementary Information

Below is the link to the electronic supplementary material.

Supplementary file1 (DOCX 1441 KB)
