# Supplementary material for: Prevalent vertebral fractures among urban-dwelling Chinese postmenopausal women: a population-based, randomized-sampling, cross-sectional study
Source: Arch Osteoporos. 2022 Sep 7;17(1):120. doi: 10.1007/s11657-022-01158-x (PMC9452427; doi:10.1007/s11657-022-01158-x)
Supplement: Supplementary file 1 — Supplementary file1 (DOCX 1441 KB) [file 11657_2022_1158_MOESM1_ESM.docx]

**Supplementary Table 1 Sample Size Calculation by Age Group based Prevalence**

| **Age group** | **Sample size** | **Assumed Prevalence** | **95% CI Lower Limit** |
| --- | --- | --- | --- |
| 50 - 54 | 44 | 5.00% | 1.00% |
| 55 - 59 | 81 | 7.00% | 3.00% |
| 60 - 64 | 100 | 12.00% | 6.00% |
| 65 - 69 | 90 | 16.00% | 10.00% |
| 70 - 74 | 80 | 20.00% | 12.00% |
| 75 - 79 | 70 | 23.00% | 15.00% |
| >80 | 53 | 37.00% | 26.00% |

| **Crude Prevalence** ^a^ | **Densitometric Osteoporosis** ^b^ | | | **NOF/IOF Clinical Diagnosis** ^d^ | | | **CSOBMR Clinical Diagnosis** ^e^ | | | **AACE Clinical Diagnosis** ^f^ | | |
| --- | --- | --- | --- | --- | --- | --- | --- | --- | --- | --- | --- | --- |
|  | n/N | % | 95% CI ^c^ | n/N | % | 95% CI | n/N | % | 95% CI | n/N | % | 95% CI |
| **Overall** | 874/2655 | 32.9 | 31.1, 34.7 | 1243/2655 | 46.8 | 44.9, 48.7 | 1084/2655 | 40.8 | 39.0, 42.7 | 1279/2655 | 48.2 | 46.3, 50.1 |
| **Age Group** |  |  |  |  |  |  |  |  |  |  |  |  |
| *<65 years* | 244/1173 | 20.8 | 18.5, 23.1 | 315/1173 | 26.9 | 24.3, 29.4 | 315/1173 | 26.9 | 24.4, 29.5 | 328/1173 | 28.0 | 25.4, 30.5 |
| *>=65 years* | 630/1482 | 42.5 | 40.0, 45.0 | 928/1482 | 62.6 | 60.2, 65.1 | 769/1482 | 51.9 | 49.3, 54.4 | 951/1482 | 64.2 | 61.7, 66.6 |
| **Geographic Region** ^g^ |  |  |  |  |  |  |  |  |  |  |  |  |
| *North China* | 338/1277 | 26.5 | 24.1, 29.0 | 527/1277 | 41.3 | 38.6, 44.0 | 430/1277 | 33.7 | 31.1, 36.3 | 538/1277 | 42.1 | 39.5, 44.9 |
| *South China* | 536/1378 | 38.9 | 36.4, 41.5 | 716/1378 | 52.0 | 49.3, 54.6 | 654/1378 | 47.5 | 44.8, 50.1 | 741/1378 | 53.8 | 51.1, 56.4 |
| **OSTA Score** |  |  |  |  |  |  |  |  |  |  |  |  |
| *Low (>-1)* | 168/1217 | 15.3 | 13.4, 17.4 | 301/1217 | 24.7 | 22.4, 27.2 | 287/1217 | 23.6 | 21.3, 26.1 | 315/1217 | 25.9 | 23.5, 28.4 |
| *Medium (-1 to -4)* | 379/970 | 39.1 | 36.1, 42.2 | 533/970 | 55.0 | 51.8, 58.1 | 466/970 | 48.0 | 44.9, 51.2 | 549/970 | 56.6 | 53.5, 59.7 |
| *High (<-4)* | 309/468 | 66.0 | 61.6, 70.2 | 409/468 | 87.4 | 84.1, 90.1 | 331/468 | 70.7 | 66.4, 74.7 | 415/468 | 88.7 | 85.5, 91.2 |
| a Categorical data are expressed as n and %; b The subject's BMD measured at the lumbar spine, the femoral neck, and the total hip. The lowest T-score was used to define osteoporosis based on the | | | | | | | | | | | | |
| World Health Organization criteria (T-score <-2.5); c 95% CI is calculated using Wilson score method; | | | | | | | | | | | | |
| d,e,f Osteoporosis by clinical diagnosis criteria is based on NOF/IOF 2014, CSOBMR, and AACE 2020 with BMD measurement and subject's self-reported information extracted from the questionnaire; | | | | | | | | | | | | |
| g Geographically divided by the Qinling Mountains-Huaihe River Line; sites locate under (above) the Line are categorized as South (North) China; | | | | | | | | | | | | |
| OEAS: Osteoporosis Evaluable Analysis Set; OSTA: Osteoporosis Self-assessment Tool for Asians; CI: confidence interval; | | | | | | | | | | | | |
| NOF/IOF: national osteoporosis foundation/international osteoporosis foundation; CSOBMR: Chinese society of bone and mineral research; AACE: American association of clinical endocrinology | | | | | | | | | | | | |

**Supplementary Table 2 Prevalence of osteoporosis (OEAS)**

**Supplementary Table 3. Univariate and multivariate logistic regression model for risk factors associated with vertebral fractures (FAS)**

| **Variable** | **Vertebral Fracture** | | | **Univariate Analysis** | | | **Multivariate Analysis** | | |
| --- | --- | --- | --- | --- | --- | --- | --- | --- | --- |
|  | **n** | **N** | **%** | **OR** | **95% CI** | **p value** | **OR** | **95% CI** | **p value** |
| **Age^** | - | - | - | 125.49 | - | <0.0001 |  |  |  |
| **BMI*** | - | - | - | 1.90 | - | 0.12 |  |  |  |
| **Region#** |  |  |  |  |  |  |  |  |  |
| East | 73 | 521 | 14.0 | - | - | 0.84 |  |  |  |
| West | 76 | 524 | 14.5 | - | - |  |  |  |  |
| South | 85 | 532 | 16.0 | - | - |  |  |  |  |
| North | 73 | 530 | 13.8 | - | - |  |  |  |  |
| Central | 81 | 527 | 15.4 | - | - |  |  |  |  |
| **Age Group**& |  |  |  |  |  |  |  |  |  |
| 50-54 | 2 | 220 | 0.9 | - | - | <0.0001 |  |  |  |
| 55-59 | 17 | 408 | 4.2 | - | - |  |  |  |  |
| 60-64 | 50 | 531 | 9.4 | - | - |  |  |  |  |
| 65-69 | 61 | 460 | 13.3 | - | - |  |  |  |  |
| 70-74 | 87 | 417 | 20.9 | - | - |  |  |  |  |
| 75-79 | 84 | 354 | 23.7 | - | - |  |  |  |  |
| 80+ | 87 | 244 | 35.7 | - | - |  |  |  |  |
| **Age >65 Years** |  |  |  |  |  |  |  |  |  |
| Yes | 319 | 1475 | 21.6 | 4.36 | 3.32, 5.73 | <0.0001 | 2.57 | 1.91, 3.48 | <0.0001 |
| No | 69 | 1159 | 6.0 |  |  |  |  |  |  |
| **Living Alone** |  |  |  |  |  |  |  |  |  |
| Yes | 80 | 542 | 14.8 | 1.00 | 0.77, 1.31 | 0.95 |  |  |  |
| No | 308 | 2087 | 14.8 |  |  |  |  |  |  |
| **Higher Education** |  |  |  |  |  |  |  |  |  |
| Yes | 55 | 409 | 13.5 | 0.88 | 0.65, 1.20 | 0.45 |  |  |  |
| No | 333 | 2222 | 15.0 |  |  |  |  |  |  |
| **Dominantly Labor Work** |  |  |  |  |  |  |  |  |  |
| Yes | 213 | 1305 | 16.3 | 1.29 | 1.04, 1.60 | 0.02 | 1.56 | 1.23, 1.99 | <0.001 |
| No | 173 | 1318 | 13.1 |  |  |  |  |  |  |
| **Lower Monthly Income** |  |  |  |  |  |  |  |  |  |
| Yes | 138 | 928 | 14.9 | 1.02 | 0.81, 1.27 | 0.88 |  |  |  |
| No | 249 | 1696 | 14.7 |  |  |  |  |  |  |
| **Smoking** |  |  |  |  |  |  |  |  |  |
| Yes | 14 | 75 | 18.7 | 1.34 | 0.74. 2.42 | 0.29 |  |  |  |
| No | 372 | 2548 | 14.6 |  |  |  |  |  |  |
| **Daily Ca or VitD** |  |  |  |  |  |  |  |  |  |
| Yes | 118 | 678 | 17.4 | 1.33 | 1.05, 1.68 | 0.02 | 1.04 | 0.79, 1.36 | 0.78 |
| No | 264 | 1929 | 13.7 |  |  |  |  |  |  |
| **Routine Physical Exercise** |  |  |  |  |  |  |  |  |  |
| Yes | 240 | 1694 | 14.2 | 0.89 | 0.71, 1.12 | 0.31 |  |  |  |
| No | 145 | 929 | 15.6 |  |  |  |  |  |  |
| **Exposure to Sunshine** |  |  |  |  |  |  |  |  |  |
| Yes | 83 | 432 | 19.2 | 1.49 | 1.14, 1.95 | 0.01 | 1.21 | 0.90, 1.63 | 0.22 |
| No | 302 | 2194 | 13.8 |  |  |  |  |  |  |
| **Sedentary Life Style** |  |  |  |  |  |  |  |  |  |
| Yes | 243 | 1611 | 15.1 | 1.10 | 0.88, 1.39 | 0.40 |  |  |  |
| No | 136 | 982 | 13.9 |  |  |  |  |  |  |
| **Waist Circumference >88cm** |  |  |  |  |  |  |  |  |  |
| Yes | 174 | 988 | 17.6 | 1.46 | 1.17, 1.82 | <0.01 | 1.45 | 1.13, 1.87 | 0.004 |
| No | 206 | 1613 | 12.8 |  |  |  |  |  |  |
| **BMI <20kg/m2** |  |  |  |  |  |  |  |  |  |
| Yes | 32 | 234 | 13.7 | 0.92 | 0.62, 1.35 | 0.74 |  |  |  |
| No | 352 | 2388 | 14.7 |  |  |  |  |  |  |
| **BMI >30kg/m2** |  |  |  |  |  |  |  |  |  |
| Yes | 40 | 202 | 19.8 | 1.49 | 1.04, 2.15 | 0.04 | 1.28 | 0.84, 1.95 | 0.26 |
| No | 344 | 2420 | 14.2 |  |  |  |  |  |  |
| **Type 2 Diabetes Mellitus** |  |  |  |  |  |  |  |  |  |
| Yes | 66 | 438 | 15.1 | 1.03 | 0.78, 1.38 | 0.77 |  |  |  |
| No | 320 | 2186 | 14.6 |  |  |  |  |  |  |
| **Cardiovascular Disease** |  |  |  |  |  |  |  |  |  |
| Yes | 200 | 1067 | 18.7 | 1.70 | 1.37, 2.11 | <0.0001 | 1.24 | 0.97, 1.59 | 0.09 |
| No | 186 | 1557 | 12.0 |  |  |  |  |  |  |
| **Menopause <40 Years** |  |  |  |  |  |  |  |  |  |
| Yes | 14 | 84 | 16.7 | 1.16 | 0.65, 2.09 | 0.51 |  |  |  |
| No | 373 | 2545 | 14.7 |  |  |  |  |  |  |
| **Surgical Menopause** |  |  |  |  |  |  |  |  |  |
| Yes | 32 | 239 | 13.4 | 0.89 | 0.60, 1.31 | 0.61 |  |  |  |
| No | 352 | 2372 | 14.8 |  |  |  |  |  |  |
| **Steroids Use** |  |  |  |  |  |  |  |  |  |
| Yes | 15 | 65 | 23.1 | 1.79 | 1.00, 1.32 | 0.07 | 1.26 | 0.66, 2.40 | 0.49 |
| No | 366 | 2552 | 14.3 |  |  |  |  |  |  |
| **HRT (estrogen/progestogen)** |  |  |  |  |  |  |  |  |  |
| Yes | 12 | 92 | 13.0 | 0.87 | 0.47, 1.62 | 0.81 |  |  |  |
| No | 371 | 2528 | 14.7 |  |  |  |  |  |  |
| **Osteoporosis Treatment**§ |  |  |  |  |  |  |  |  |  |
| Yes | 163 | 795 | 20.5 | 1.87 | 1.50, 2.33 | <0.0001 | 1.93 | 1.29, 2.90 | 0.002 |
| No | 222 | 1830 | 12.1 |  |  |  |  |  |  |
| **Sufficient Osteoporosis Treatment**§ | |  |  |  |  |  |  |  |  |
| Yes | 51 | 177 | 28.8 | 2.56 | 1.81, 3.62 | <0.0001 | - | - | - |
| No | 334 | 2448 | 13.6 |  |  |  |  |  |  |
| **Height Decreased** |  |  |  |  |  |  |  |  |  |
| Yes | 182 | 819 | 22.2 | 2.22 | 1.78, 2.76 | <0.0001 | 1.36 | 1.05, 1.75 | 0.02 |
| No | 204 | 1788 | 11.4 |  |  |  |  |  |  |
| **Fall** |  |  |  |  |  |  |  |  |  |
| Yes | 186 | 774 | 24.0 | 2.60 | 2.09, 3.25 | <0.0001 | 1.78 | 1.38, 2.30 | <0.0001 |
| No | 201 | 1856 | 10.8 |  |  |  |  |  |  |
| **HIP Fracture** |  |  |  |  |  |  |  |  |  |
| Yes | 15 | 34 | 44.1 | 4.71 | 2.37, 9.36 | <0.001 | 2.28 | 1.09, 4.76 | 0.03 |
| No | 373 | 2600 | 14.4 |  |  |  |  |  |  |
| **NVNH Fractures** |  |  |  |  |  |  |  |  |  |
| Yes | 74 | 388 | 19.1 | 1.45 | 1.09, 1.92 | 0.01 | 0.89 | 0.65, 1.23 | 0.49 |
| No | 314 | 2244 | 14.0 |  |  |  |  |  |  |
| **Bone Health Awareness** |  |  |  |  |  |  |  |  |  |
| Yes | 126 | 843 | 15.0 | 1.05 | 0.84, 1.33 | 0.64 |  |  |  |
| No | 247 | 1728 | 14.3 |  |  |  |  |  |  |
| **Osteoporosis by DXA BMD** |  |  |  |  |  |  |  |  |  |
| Yes | 210 | 868 | 24.2 | 2.84 | 2.28, 3.54 | <0.0001 | 2.52 | 1.96, 3.22 | <0.0001 |
| No | 178 | 1764 | 10.1 |  |  |  |  |  |  |
| Categorical data are expressed as n, N, and %; n=the number of subjects with vertebral fractures, N=the number of subjects included in the analysis on | | | | | | | | | |
| the outcome of interest; %=the percentage of subjects with vertebral fractures among all subjects included in the outcome of interest | | | | | | | | | |
| All risk factors (independent variable) are binary for vertebral fractures odds of yes versus no; 95% CI is based on normal approximations | | | | | | | | | |
| Age as continuous variable and age groups as ordinal variable are not included in multivariate analysis; | | | | | | | | | |
| ^ unit OR: 1.10; *unit OR: 1.02; #Cochran Armitage Trend Test p=0.69; &Cochran Armitage Trend Test p<0.0001 | | | | | | | | | |
| § Infomration on osteoporosis treatment is given by subjects who interviewed the investigators; sufficient osteoporosis treatment is defined as | | | | | | | | | |
| antiresorptive treatment and/or vitamin D +/- calcium, or teriparatide for at least 12 months; sufficient osteoporosis treatment is not included in | | | | | | | | | |
| multivariate analysis due to its potential colinearity with osteoporosis treatment; OR: odds ratio; CI: confidence interval | | | | | | | | | |

**
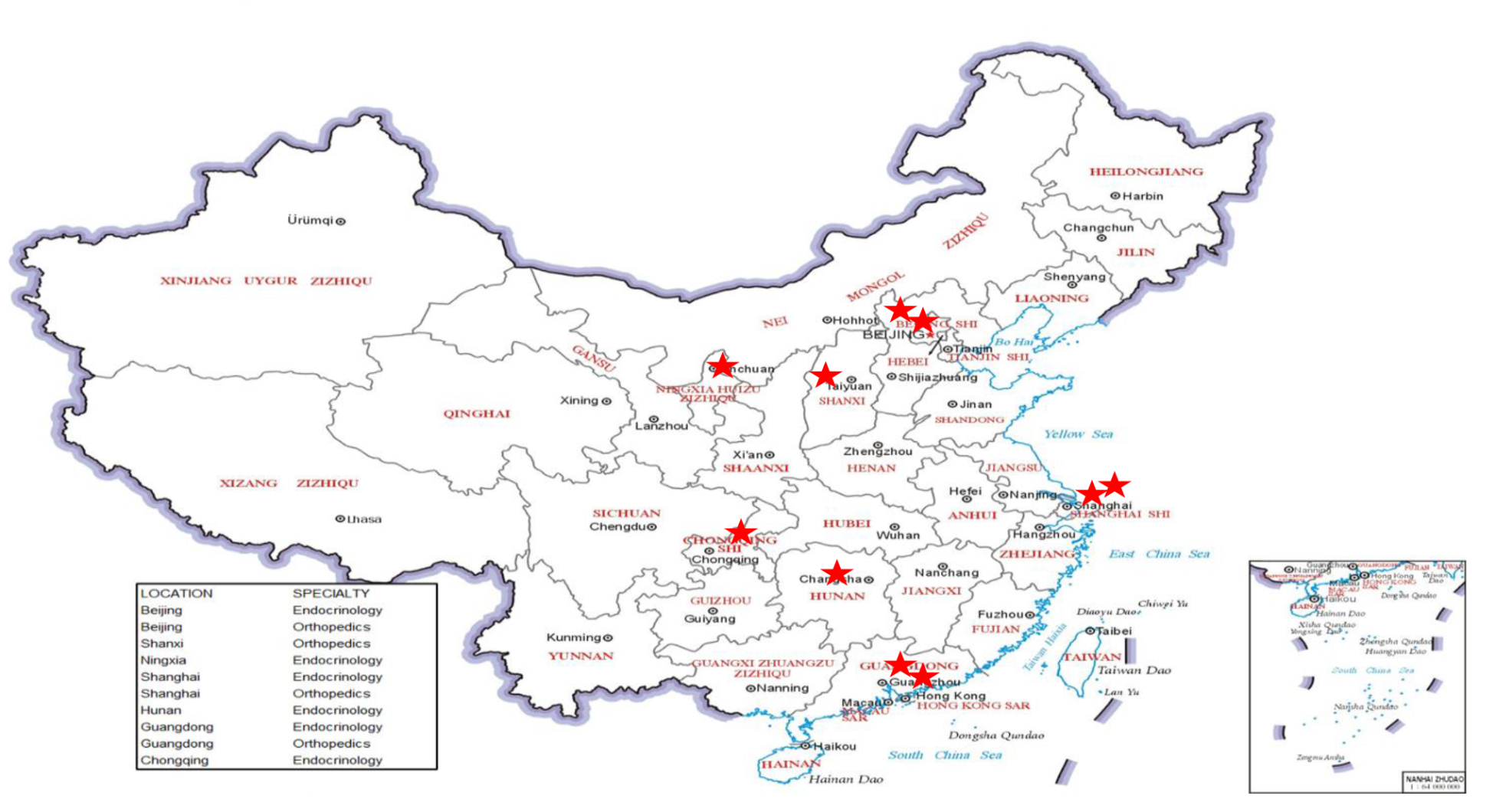
**

**Supplementary Figure 1 The study site's medical specialty and geographic distribution**


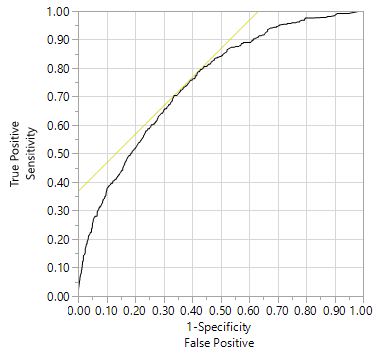


**AUC: 0.7496**

**Supplementary Figure 2 Receiver operating characteristic area under the curve for logistic regression model identifying VF risks**
